# Characterization of Novel P-Selectin Targeted Complement Inhibitors in Murine Models of Hindlimb Injury and Transplantation

**DOI:** 10.3389/fimmu.2021.785229

**Published:** 2021-11-25

**Authors:** Chaowen Zheng, Jerec Ricci, Qinqin Zhang, Ali Alawieh, Xiaofeng Yang, Satish Nadig, Songqing He, Pablo Engel, Junfei Jin, Carl Atkinson, Stephen Tomlinson

**Affiliations:** ^1^ Department of Microbiology and Immunology, Medical University of South Carolina, Charleston, SC, United States; ^2^ Division of Hepatobiliary Surgery, The First Affiliated Hospital of Guangxi Medical University, Nanning, China; ^3^ The Lee Patterson Allen Transplant Immunobiology Laboratory, Department of Transplant Surgery, Department of Surgery, Medical University of South Carolina, Charleston, SC, United States; ^4^ Department of Surgery, Medical University of South Carolina, Charleston, SC, United States; ^5^ Department of Thyroid and Breast Surgery, Nanxishan Hospital of Guangxi Zhuang Autonomous Region, Guilin, China; ^6^ Department of Biomedical Sciences, University of Barcelona, Barcelona, Spain; ^7^ Guangxi Key Laboratory of Molecular Medicine in Liver Injury and Repair, The Affiliated Hospital of Guilin Medical University, Guilin, China; ^8^ Department of Pulmonary Medicine, University of Florida, Gainesville, FL, United States; ^9^ Ralph H. Johnson Veteran Affairs Medical Center, Charleston, SC, United States

**Keywords:** complement, P-selectin, ischemia reperfusion injury, hind limb, vascularized composite allotransplantation, targeted therapeutic

## Abstract

The complement system has long been recognized as a potential druggable target for a variety of inflammatory conditions. Very few complement inhibitors have been approved for clinical use, but a great number are in clinical development, nearly all of which systemically inhibit complement. There are benefits of targeting complement inhibition to sites of activation/disease in terms of efficacy and safety, and here we describe P-selectin targeted complement inhibitors, with and without a dual function of directly blocking P-selectin-mediated cell-adhesion. The constructs are characterized *in vitro* and in murine models of hindlimb ischemia/reperfusion injury and hindlimb transplantation. Both constructs specifically targeted to reperfused hindlimb and provided protection in the hindlimb ischemia/reperfusion injury model. The P-selectin blocking construct was the more efficacious, which correlated with less myeloid cell infiltration, but with similarly reduced levels of complement deposition. The blocking construct also improved tissue perfusion and, unlike the nonblocking construct, inhibited coagulation, raising the possibility of differential application of each construct, such as in thrombotic *vs*. hemorrhagic conditions. Similar outcomes were obtained with the blocking construct following vascularized composite graft transplantation, and treatment also significantly increased graft survival. This is outcome may be particularly pertinent in the context of vascularized composite allograft transplantation, since reduced ischemia reperfusion injury is linked to a less rigorous alloimmune response that may translate to the requirement of a less aggressive immunosuppressive regime for this normally nonlife-threatening procedure. In summary, we describe a new generation of targeted complement inhibitor with multi-functionality that includes targeting to vascular injury, P-selectin blockade, complement inhibition and anti-thrombotic activity. The constructs described also bound to both mouse and human P-selectin which may facilitate potential translation.

## Introdution

Reperfusion of hypoxic/ischemic tissue, that occurs either naturally, as a result of an intervention, or following transplantation, can result in cellular injury. Ischemia reperfusion (IR) injury (IRI) is a component of multiple pathological conditions such as myocardial infarction, ischemic stroke, physical trauma, and it is also an unavoidable consequence of organ or tissue transplantation. In the current study we describe the development of novel site-targeted anti-inflammatory molecules, and their characterization in murine hindlimb IRI that models either acute limb warm ischemia/reperfusion or vascularized composite allograft (VCA) (hindlimb) transplantation that incorporates cold ischemia.

Acute limb ischemia occurs when there is a sudden decrease in limb perfusion, and is associated with high rates of mortality and amputation. It affects various tissue compartments, including the skin, muscle and peripheral nervous system. Common etiologies include arterial embolism, *in situ* thrombosis of an atherosclerotic artery, and trauma (1). With regard to the hindlimb transplantation model, VCA transplantation has the potential to benefit a wide range of patients including those with congenital anomalies, traumatic injuries, or those needing complex reconstruction following tumor resection. However, the clinical benefit to patients remains limited due to the requirement of high-dose, lifelong and multidrug immunosuppression, together with the fact that the procedure is usually life-improving rather than life-saving. While many conventional immunosuppressive regimens exist that target the adaptive immune system, few therapies exist that target early innate mechanisms of graft injury that are linked to the vigor of a subsequent alloimmune response. One such type of early graft injury is IRI, an unavoidable consequence of transplantation.

Both complement activation and neutrophils have been shown to play important roles in IRI, and endothelial expression of the P-selectin adhesion molecule is important for neutrophil recruitment [for reviews, see (2, 3)]. Complement activation products that have been shown to play a role in IRI are the anaphylatoxins (C3a and C5a) that can recruit and activate immune cells, and the proinflammatory and cytolytic membrane attack complex (4). Both complement inhibition and P-selectin blockade have independently been shown to be protective in various models of IRI. Furthermore, complement activation products can upregulate P-selectin expression, and P-selectin can directly activate complement (5–9). Here we describe the development and characterization of a complement inhibitor that specifically targets to P-selectin. We linked the murine complement inhibitor, Crry, to one of two different anti-P-selectin single chain antibody (scFv) targeting vehicles that we derived from monoclonal antibodies – one which blocked P-selectin leukocyte adhesion function, and one that did not. The Crry fusion partner has the structural and combined functional properties of human decay-accelerating factor and membrane cofactor protein, and inhibits all complement pathways at the C3 activation step (10). The constructs are thus expected to target *via* P-selectin upregulation at sites of inflammation, and one construct is expected to be bi-functional in that it both directly blocks P-selectin function and inhibits complement.

## Materials and Methods

### Construction of Plasmids and Expression of Recombinant Proteins

For construction of B.PSel scFv and NB.PSel scFv, total RNA was isolated from anti-P-selectin hybridoma cell lines 2.12 and 2.3, respectively, as described (11). The 2.12 hybridoma produces a mAb that blocks P-selectin adhesion function, whereas the mAb from the 2.3 hybridoma is non-blocking (12). Using methods we have described (13), cDNA corresponding to mRNA was synthesized, with primers for variable heavy (VH) and variable light (VL) chain domains. PCR products were cloned and positive colonies sequenced and aligned using NCBI blast database. A second round of PCR was subsequently conducted to amplify VH and VL chain cDNA, and a CD5 signal peptide sequence and a His tag (6X) sequence added to the 5’ end of the VH chain, and a (Gly_4_Ser_1_)_3_ sequence was inserted between the VH and VL fragments. For construction of the B.PSel-Crry and NB.PSel-Crry expression plasmids, each scFv sequence was linked to the sequence encoding the extracellular region of mouse Crry (residues 1-319, Genbank accession number NM013499) by overlapping PCR with the inclusion of a (G_4_S_2_)_2_ linker sequence. The fusion constructs were cloned into the pEE12.4 expression vector (Lonza), and expressed in Expi293 cells as described by the provider (ThermoFisher). After harvest, the supernatant was filtered (0.22 μm) and protein purified using a His60 Nickle column (Clontech) and subsequent filtration with 30 kDa MW cut-off with PBS buffer exchange. Proteins were stored at -80C, and once thawed stored at 4C for up to 2 weeks. To note, we were unable to express the scFv constructs alone (ie without fusion partner).

### *In Vitro* Characterization of Recombinant Proteins

P-selectin binding of fusion proteins was confirmed by standard ELISA using recombinant mouse P-selectin-Ig (BD Biosciences) as capture antigen and anti-His tag Ab (Clontech) for detection (data not shown). Binding characteristics were analyzed by surface plasmon resonance (SPR). For SPR binding assays, binding affinity (KD) and kinetic parameters ((Ka and Kd) were determined using a recombinant mouse P-selectin ligand (SinoBiological) printed onto a bare gold-coated (47 nm) PlexArray Nanocapture Sensor Chip (Plexera Bioscience). Analyses were performed by Creative Peptides (Shirley, NY) with real-time binding signals recorded and analyzed by Data Analysis Module (DAM, Plexera, Bioscience, Seattle, WA), and kinetic analysis performed using BIAevaluation 4.1 software (Biacore, Inc.). Complement inhibitory activity of the constructs was measured following purification and before each use by flow cytometric analysis of complement deposition in a standard zymosan assay (14).

### Hindlimb IRI

Adult male C57BL/6 mice (Jackson Laboratory) aged 8-10 weeks and weighing 20-25 g were anesthetized with 7.5 mg/kg ketamine and 10 mg/kg xylazine by i.p. injection. Animal respiration was continuously monitored and body heat maintained with a heating pad. A 1/8 inch 4.5 oz orthodontic rubber band was placed around the right hindlimb for 2 hours of ischemia before reperfusion (15). Upon reperfusion, mice were injected with 0.1 ml vehicle as control (PBS) or with indicated dose of NB.Psel-Crry or B.Psel-Crry in 0.1 ml PBS *via* tail vein. Mice were allowed to recover from anesthesia under a heating lamp. At 24 hours post-reperfusion, mice were euthanized and blood and hindlimb tissue collected.

### Hindlimb Transplantation

Male C57BL/6J mice at 10–12 weeks of age (Jackson Laboratory) were used as both donor and recipient in a vascularized composite isograft (VCI) transplantation model. For vascularized composite allograft (VCA) transplantation used for survival study, male Balb/c donors and male C57BL/6J recipients (10–12 weeks) were used. The procedure was perfomed as described (16), except that following transection of muscle and bone (in both donors and recipients) the bone marrow was packed with Surgicel Fibrillar (Ethicon) for hemostasis. Animals were anesthetized with 7.5 mg/kg ketamine and 10 mg/kg xylazine by i.p. injection. Following transplantation, mice were evaluated daily with macroscopic inspection of the hindlimb, weight measurement, and perfusion using laser speckle perfusion Doppler and conventional laser Doppler. Graft survival was determined by visualization of rejection (skin erythema and necrosis, hair loss) as previously described (17). All animal experiments were conducted in accordance with the Guide for the Care and Use of Laboratory Animals of the National Institutes of Health and following the Institutional Animal Care and Use Committee (IACUC) protocols authorized by the Medical University of South Carolina, Charleston, SC.

### Measurement of Hindlimb Blood Flow

Superficial hindlimb blood flow was monitored in animals anesthetized with 1% isoflurane using a laser speckle contrast imager (moor-FLPI2, Moor Instruments) and a conventional laser Doppler monitor (moorVMS-LDF, Moor Instruments). Images were analyzed with moorFLPI-2 review V5.0. Measurement in the hindlimb IRI and VCI transplantation models were taken pre ligation/surgery and at indicated times post reperfusion. For both methods, flow measurements were obtained as a perfusion ratio at the popliteal artery using the formula ischemic limb/non-ischemic limb.

### Bleeding Time and Volume

Tail bleed time in 10-12 week old male C57BL/6J mice (n = 5) was measured 2 hours after injection of PBS, B.PSel-Crry or NB.PSel-Crry, as described (18). Briefly, anesthetized animals were placed in prone position and a distal 5mm segment of the tail amputated. The tail was immediately immersed in pre-warmed isotonic saline at 37°C and each animal monitored for 20 minutes, even if bleeding ceased in order to detect any re-bleeding. If bleeding on/off cycles occurred, the sum of bleeding times within the 20-minute period was used. Body weight, including the tail tip, was measured again, and the volume of blood loss during the experimental period estimated from the reduction in body weight.

### Fluorescence Tomography and Live Animal Imaging

B.Psel-Crry and NB.Psel-Crry were labeled using Xenolight CF 750 NIR Fluorescent Dye according to manufacturer’s instructions (Perkin Elmer). After 2 hours of hindlimb ischemia and upon reperfusion, 0.25 mg fluoresectently labeled NB.Psel-Crry or B.Psel-Crry (or PBS control) was injected *via* the tail vein. At 24 hrs post-reperfusion, mice were anesthetized and imaged laterally using a Maestro EX imaging system (Perkin Elmer). Fluorescent signal was quantified using supplied software.

### Histopathology and Immunomicroscopy

Tissue specimens were taken from de-boned hindlimb samples and either frozen in liquid nitrogen and placed in -80°C, or fixed in 10% formalin at 4°C overnight and subsequently processed to paraffin. Sections were stained with Hematoxylin and Eosin (H&E) and scored independently by two pathologists blinded to treatments. Tissue sections were evaluated for muscle fiber injury as previously described (19), and scored based on percentage of injured fibers from the total number of muscle fibers counted in each tissue sample. C3d immunohistochemistry (IHC) staining was performed as described (20), and deposition quantified by calculating the mean grey intensity in ImageJ software (NIH). Myeloperoxidase (MPO) IHC staining was performed as described (21), and quantified by counting the number of MPO-positive cells per 400x field. P-selectin was detected in paraffin-embedded sections (4 um) by IHC using anti-P-selectin antibody (ab255822, Abcam) as described (22), and signal intensity quantified after automated random field sampling by Image J software. All microscopic examinations and quantifications were carried out in a blinded fashion.

### Pharmacokinetics

To determine the circulatory half-life of each construct, 0.5 mg NB.Psel-Crry or B.Psel-Crry was injected *via* tail vein, and blood collected for serum preparation at 0, 15min, 30 min, 1 h, 2 h, 4 h, 12 h, 24 h and 48 h. Plasma concentration of each construct was determined by anti-Crry ELISA using corresponding purified construct as standard, as described (23).

### Statistical Analysis

All data are presented as mean ± SD. All data were subjected to statistical analysis using Prism software version 8 (GraphPad Software Company). Statistical analyses of the data were interpreted by non-parametric One-Way ANOVA (Kruskal-Wallis) for ordinal data with Dunn’s correction for multiple comparisons. Parametric One-Way ANOVA was used for continuous variables. When comparing each group to one group, Dunnett’s correction for multiple comparisons was used. When comparing each group to every other group, Tukey’s correction was used. Mantel-Cox (log-rank) test was used to compare the survival distributions of two groups. T test was used when only two groups were compared. A p value of less than 0.05 was considered significant.

## Results

### *In Vitro* Characterization of PSel-Crry Constructs

To target a complement inhibitor to sites of P-selectin expression, the extracellular region of murine Crry was linked to an anti-P-selectin scFv construct derived from either a blocking or non-blocking (with regard P-selectin/PSGL-1 interaction) anti-P-selectin IgG mAb (see methods). The blocking and non-blocking constructs are designated B.PSel-Crry and NB.PSel-Crry, respectively. The purified constructs had the expected MW of 62 kDa and were >95% pure as determined by SDS-PAGE (not shown). Both constructs retained parent IgG mAb binding specificity as determined by their ability to bind to P-selectin by ELISA (not shown) and by surface plasmon resonance (SPR) (**Supplementary Figure 1**). Binding characteristics analyzed by SPR demonstrated similar and moderate binding affinity, with similar kinetic parameters. The complement inhibitory activity of Crry was retained in the fusion proteins, as demonstrated by a dose-dependent inhibition of C3 deposition on zymosan beads, with each construct exhibiting efficacies that were not significantly different (**Supplementary Figure 2A**). As do the parent mAbs from which the scFv’s were derived, both constructs also bound to human P-selectin (**Supplementary Figure 2B**).

### *In Vivo* Targeting and Circulatory Half-Life of PSel-Crry Constructs

**Figure 1:**
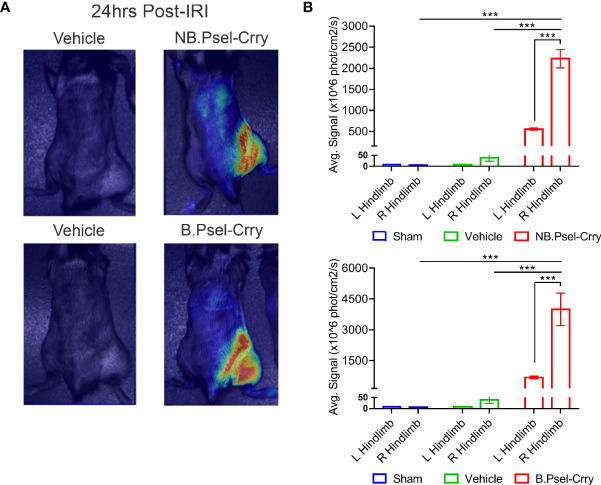
B.PSel-Crry and NB.PSel-Crry specifically traffic to hindlimb subjected to ischemia and reperfusion. Fluorescently labeled construct (0.25 mg) was administered i.v. to mice upon reperfusion after 2 hours of ischemia. At 24 h post-administration, mice were imaged with a near infrared Maestro device at 24hrs post-administration. Live animal fluorescence tomography images were taken at 24 h after construct administration. **(A)** Representative whole body images. **(B)** Quantification of fluorescence intensity in ipsi and contralateral hindlimbs. Dunnett’s correction for multiple comparisons. Quantification data expressed as Mean ± SD, ***p ≤ 0.001. n = 4.

P-selectin is upregulated in mouse hindlimbs following hindlimb IR (24) (and as shown below). To confirm that both constructs target appropriately *in vivo*, we analyzed whole body distribution of fluorescently labeled constructs by live animal imaging. Two hrs after hindlimb ischemia and upon reperfusion, fluorescently labeled B.Psel-Crry or NB.Psel-Crry was injected *via* the tail vein. Fluorescence tomography of live animals was performed 24 hr later to determine the biodistribution of each construct. Both B.Psel-Crry and NB.Psel-Crry specifically localized to the ischemic/reperfused (right) limb (**Figure 1**). There was low level fluorescence associated with the contralateral limb and with the position of lungs (not quantified), which is consistent with sites of secondary injury in this model (25). Thus, there was retention of the construct at the target site, and for an optimum design feature this would be accompanied by a short circulatory half-life to minimize effect on systemic complement inhibition. Following injection of a 0.5 mg dose, serum concentration of the constructs was between 4-6 ug/ml measured at 15 min. Both constructs had a two-phase elimination profile with a T_1/2_ fast of 0.9h and 0.73h, and a T_1/2_ slow of 30.6h and 26h for B.PSel-Crry and NB.PSel-Crry, respectively, and which were not significantly different (**Supplementary Figure 3**). Over 75% of the peak serum concentration of drug was eliminated within 5 h. The early elimination profiles likely reflect a rapid tissue redistribution

### PSel-Crry Constructs Reduce Hindlimb IRI and Inflammatory Markers

**Figure 2:**
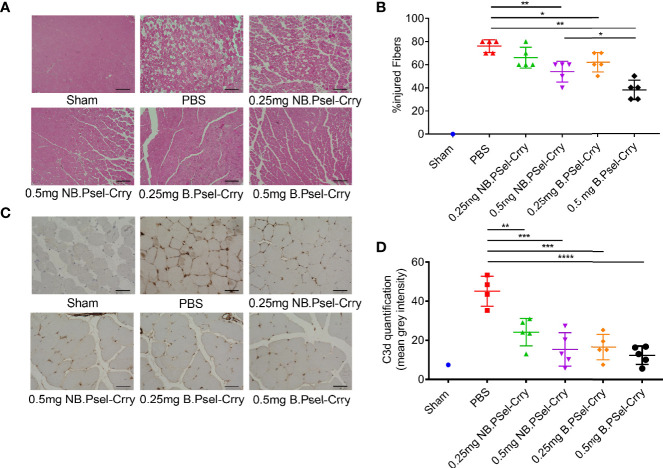
B.PSel-Crry and NB.PSel-Crry reduce injury after hindlimb ischemia and reperfusion. A murine hindlimb IRI model was used to evaluate the effect of B.PSel and NB.PSel administered i.v. upon reperfusion after 2 h ischemia. **(A)** Representative H&E stained muscle sections revealed reduced skeletal muscle injury, edema, and myeloid cell infiltrate in mice treated with B.PSel or NB.PSel as compared to the vehicle control (PBS). Scale bars, 200μm. **(B)** Quantification of IRI, showing reduction in histology injury scores with both constructs. At higher dose of 0.5 mg, B.PSel-Crry was significantly more protective than NB.PSel-Crry. No injury was seen in the sham injected mice. Non-parametric One-Way ANOVA (Kruskal-Wallis) with Dunn’s correction. **(C)** Representative images of sections stained for C3d deposition. Scale bars, 50μm. **(D)** Quantification of C3d deposition. Parametric One-Way ANOVA with Tukey’s correction. Quantification data expressed as Mean ± SD, *p ≤ 0.05, **p ≤ 0.01, ***p ≤ 0.001, ****p ≤ 0.0001. n = 5 for all groups.

**Figure 3:**
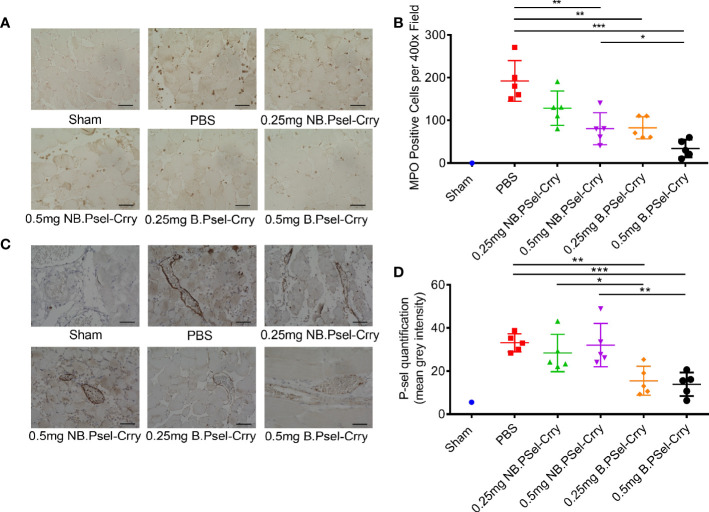
B.PSel-Crry and NB.PSel-Crry reduce inflammation markers after hindlimb ischemia and reperfusion. A murine hindlimb IRI model was used to evaluate the effect of B.PSel and NB.PSel administered i.v. upon reperfusion after 2 h ischemia. **(A)** Representative images of sections stained for MPO. At the higher dose of 0.5 mg, there were lower numbers of infiltrating myeloid cells in mice treated with B.PSel-Crry compared to NB.PSel-Crry. Scale bars, 50μm. **(B)** Quantification of infiltrating myeloid cells performed by assessing the number of MPO-positive cells per field. Parametric One-way ANOVA with Tukey’s correction. **(C)** Representative images of sections stained for P-selectin. Scale bars, 50μm. **(D)** Quantification of P-selectin expression, which was significantly lower in B.PSel-Crry treated mice compared to NB.PSel-Crry treated mice at both concentrations. Parametric One-Way ANOVA with Tukey’s correction. Quantification data expressed as Mean ± SD, *p ≤ 0.05, **p ≤ 0.01, ***p ≤ 0.001. n = 5 for all groups.

Following hindlimb IR, animals were treated intravenously with vehicle or with either B.Psel-Crry or NB.Psel-Crry at indicated concentrations. At 24 hr after reperfusion, hindlimb tissue was isolated and processed for analysis. Histopathological analysis revealed that compared to vehicle treated controls, both NB.PSel-Crry and B.PSel-Crry treatment reduced edema and polymorphonuclear infiltrates (**Figures 2A, B**). Furthermore, the effect of each inhibitor was dose-dependent, and at the higher dose of 0.5 mg, B.Psel-Crry provided better protection than NB.PSel-Crry. No edema or polymorphonuclear infiltrates were observed in hindlimbs from sham operated mice. Correlating with histopathological injury, both constructs reduced C3d deposition in reperfused hindlimbs, and there was no difference in C3d deposition between the blocking and nonblocking construct at each dose (**Figures 2C, D**). To address any differences in the *in vivo* P-selectin blocking *vs*. nonblocking characteristics of the constructs, we performed anti-MPO immunohistochemistry on hindlimb sections. The enzyme MPO is abundantly expressed in myeloid cells, and particularly neutrophils, and serves as a marker of their presence within tissue specimens. A dose-dependent reduction in MPO-positive cells was observed following treatment with either NB.PSel-Crry or B.PSel-Crry, but there was significantly less infiltration with B.PSel-Crry compared to NB.Psel-Crry at the more protective higher 0.5 mg dose (**Figures 3A, B**). B.PSel-Crry, but not NB.Psel-Crry, also reduced post-IR P-selectin expression (**Figures 3C, D**).

### Effect of Constructs on Bleeding Time and Hindlimb Perfusion Following IRI

**Figure 4:**
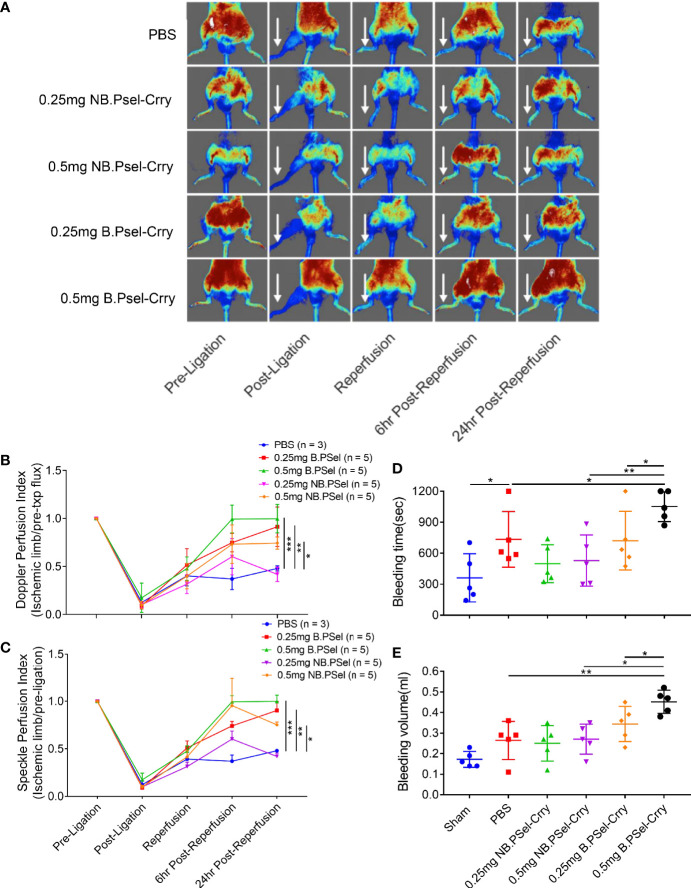
Effect of B.PSel-Crry and NB.PSel-Crry on hindlimb perfusion following ischemia/reperfusion and on bleeding time and volume. Mice were injected with construct or PBS upon reperfusion after 2 h ischemia and hindlimb perfusion measured by laser Doppler flow of laser speckle imaging at indicated times. **(A)** Representative laser speckle doppler images from each treatment group, with arrows indicating ligated hindlimb. **(B)** Quantification of perfusion by laser Doppler flow using a single point on each paw and normalizing to pre-ligation value. Parametric One-Way ANOVA with Tukey’s correction. **(C)** Quantification of perfusion by laser speckle doppler using a point on each paw and normalizing to pre-ligation value. Parametric One-Way ANOVA with Tukey’s correction. Quantification data expressed as Mean ± SD; *p ≤ 0.05, **p ≤ 0.01, ***p ≤ 0.001. n = 5 for all groups. For bleeding determinations, indicated dose of construct or PBS administered *via* i.v. injection, and mice monitored over a 20 min. period after tail clip. **(D)** Tail bleed time. Bleeding time was significantly longer in mice treated with 0.5 mg dose of B.PSel-Crry. Parametric One-Way ANOVA with Tukey’s correction. **(E)** Tail bleed volume, which was significantly larger in mice treated with 0.5 mg dose of B.PSel-Crry. Parametric One-Way ANOVA with Tukey’s correction. Quantification data expressed as Mean ± SD; *p ≤ 0.05, **p ≤ 0.01. n = 5.

Since P-selectin plays important roles in platelet trafficking and coagulation, we investigated the effect of each construct on bleeding time and volume using a tail clip assay to investigate platelet hemostatic function, and on tissue perfusion after hindlimb IR using doppler flow and laser speckle imaging. The NB.PSel-Crry construct had no effect on bleeding time or volume, whereas at the higher 0.5 mg dose, B.PSel-Crry increased both bleeding time and volume compared to the nonblocking construct and vehicle (**Figures 4D, E**). To assess tissue perfusion after hindlimb IR, we used the same ischemia and treatment protocol as in the studies above, with acquisition of both doppler and laser speckle measurements at 6- and 24-hours post-reperfusion. For doppler flow determinations, a single point on each paw representing the area of the saphenous artery was measured. Both doppler and speckle determinations showed that B.PSel-Crry at both low (0.25mg) and high (0.5mg) dose improved tissue perfusion measured at 6 and 24 hours after reperfusion compared to vehicle. The nonblocking construct improved tissue perfusion only at the higher 0.5 mg dose (**Figures 4A–C**).

### B.Psel-Crry Characterization in an IRI Model of Vascularized Composite Isograft Orthotopic Hindlimb Transplantation

Based on the superior efficacy of B.Psel-Crry *vs*. NB.PSel-Crry in a hindlimb IRI model, we investigated the effect of B.PSel-Crry (at both 0.25mg and 0.5 mg dose) on graft inflammation and IRI in a vascularized composite isograft (VCI) model of orthotopic hindlimb transplantation. We employed an isograft model to avoid any potential confounding effects of an early alloimmune response, and analyzed graft muscle tissue at 6 and 24 h post-transplant.

**Figure 5:**
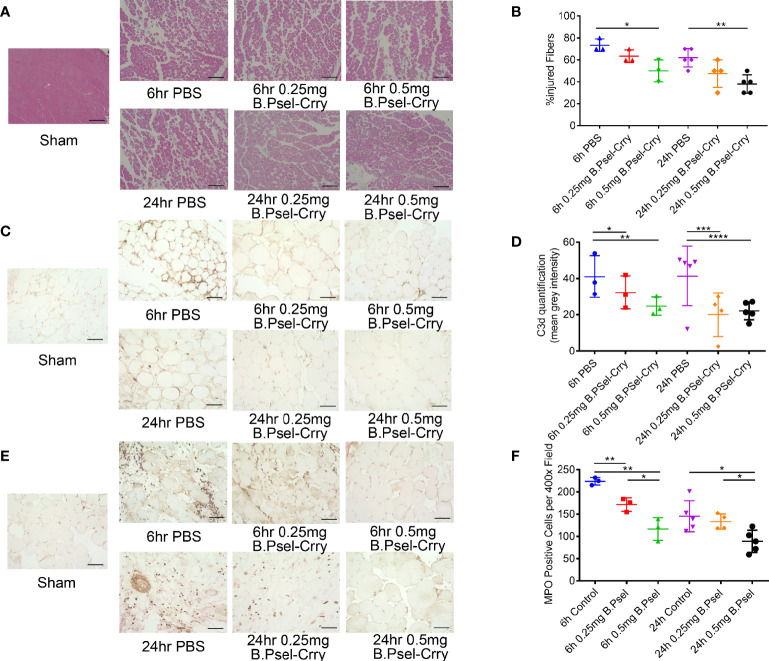
B.PSel-Crry reduces hindlimb graft injury and inflammation following vascularized composite isograft transplantation. Tissue sections from the thigh muscle of hindlimb grafts isolated at either 6 h or 24 h post-transplant were prepared. **(A)** Representative H&E stained muscle sections demonstrating muscle necrosis and myeloid cells infiltrates in controls that are largely absent in 0.5mg B.PSel-Crry treated mice. Scale bars, 200μm. **(B)** Quantification of injury shown as the percentage of injured fibers from the total number of muscle fibers counted in each tissue sample. Non-parametric One-Way ANOVA (Kruskal-Wallis) with Dunn’s correction. **(C)** Representative images of sections stained for C3d deposition. Scale bars, 50μm. **(D)** Quantification of C3d staining by measuring mean fluorescence intensity, showing a dose-dependent reduction in C3d by B.Psel-Crry treatment measured at both 6 hours and 24 hours. Parametric One-Way ANOVA with Tukey’s correction. **(E)** Representative images of sections stained for MPO. Scale bars, 50μm. **(F)** MPO quantification represented as number of MPO positive cells per field, showing a dose-dependent reduction in MPO by B.Psel-Crry treatment measured at both 6 hours and 24 hours. Parametric One-Way ANOVA with Tukey’s correction. Quantification data expressed as Mean ± SD; *p ≤ 0.05, **p ≤ 0.01, ***p ≤ 0.001, ****p ≤ 0.0001.

Histopathological analysis of graft muscle measured at 24 hr post-transplant revealed that a 0.5 mg dose of B.PSel-Crry administered to the recipient reduced edema, necrosis and polymorphonuclear infiltrates as compared to graft muscle from PBS treated controls (**Figures 5A, B**). There were no significant differences in injury scores at 6 h post-transplant and at the lower dose, as compared to PBS treated controls. C3d deposition, however, was reduced by B.PSel-Crry treatment at both doses when measured at both 6 and 24 h post-transplant (**Figures 5C, D**). B.PSel-Crry also reduced MPO-positive cell infiltration at both doses and at both time points, with the exception of the low dose B.PSel-Crry measured at 24 h post-transplant (**Figures 5E, F**).

### B.PSel-Crry Improves Graft Perfusion Following Vascularized Composite Isograft Transplantation

**Figure 6:**
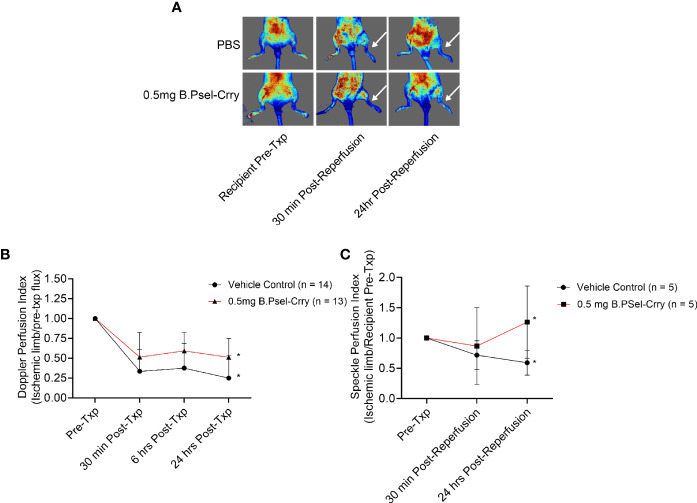
B.PSel-Crry improves hindlimb perfusion at 24 hours post-transplantation in a vascularized composite isograft. Hindlimb transplanted recipients were treated immediately post-reperfusion as indicated and perfusion measured in the graft paw by conventional laser Doppler flow and laser speckle Doppler imaging at indicated time points after transplantation. **(A)** Representative Laser speckle Doppler images. **(B)** Quantification of perfusion by conventional laser Doppler flow. T-test. **(C)** Quantification of perfusion by Laser speckle doppler. T-test. Perfusion was significantly improved at 24 hours post-transplantation following 0.5mg B.PSel-Crry treatment. Data expressed as Mean ± SD; *p ≤ 0.05. n = as indicated in figure.

Data above show that B.Psel-Crry improves tissue perfusion following hindlimb ischemia in model of warm IRI. Here, we treated recipients of a hindlimb isograft with 0.5 mg B.PSel-Crry and measured graft perfusion by laser Doppler flow and laser speckle imaging. Perfusion measured by both techniques was improved in B.PSel-Crry treated recipients by 24 h post-transplant, although not at earlier time points, as compared to PBS treated recipients (**Figure 6**).

### B.PSel-Crry Prolongs Survival of Hindlimb Vascularized Composite Allografts

**Figure 7:**
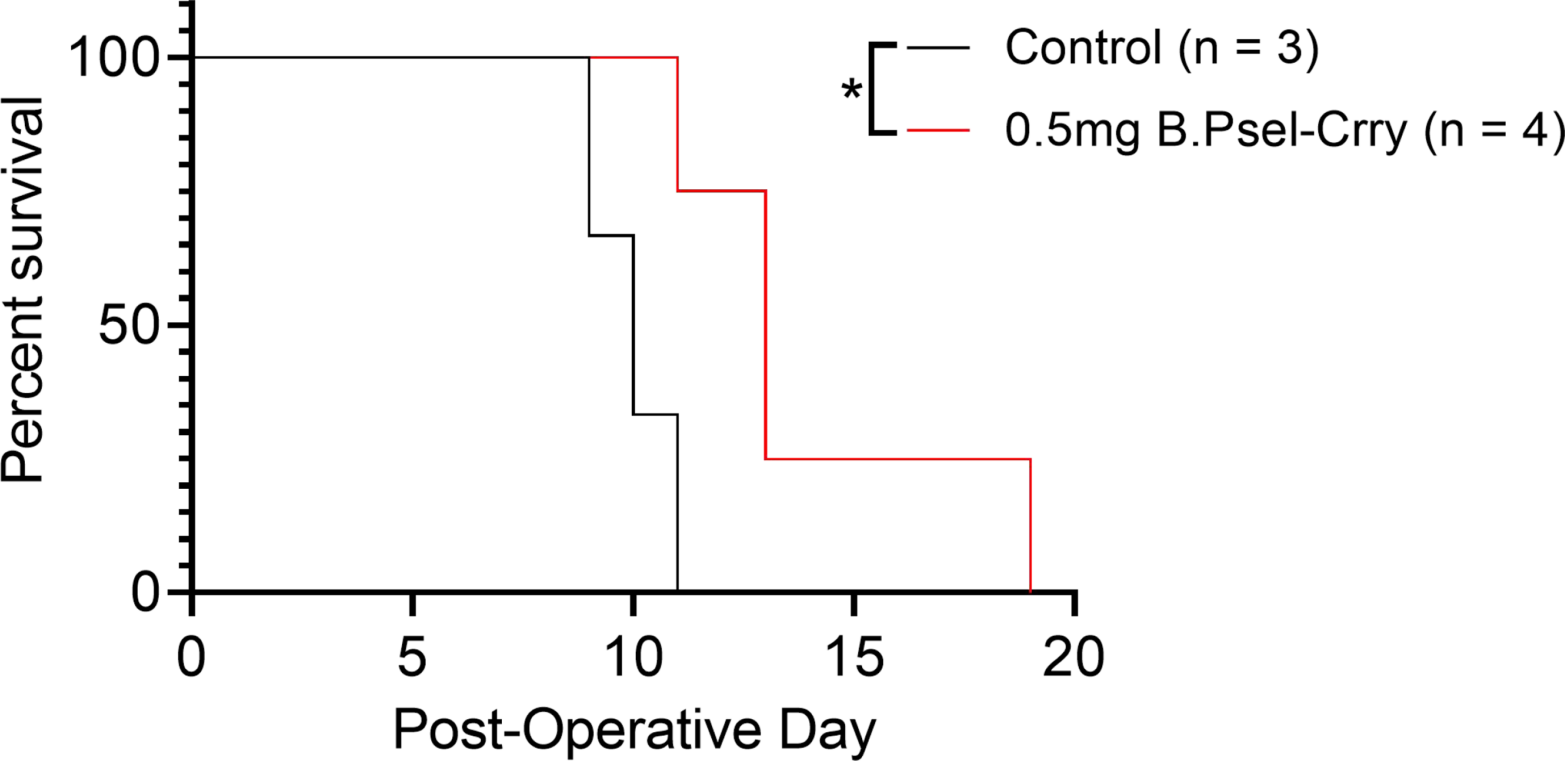
B.PSel-Crry improves survival of hindlimb vascularized composite allografts. Allograft survival for orthotopic hindlimb VCA mice was classified as days until Banff clinical grade 4 rejection was reached. Graft survival curve comparing PBS and B.PSel-Crry treated groups. A single postoperative dose of 0.5mg B.PSel-Crry significantly improved hindlimb allograft survival. Mantel-Cox (log-rank) test. *p ≤ 0.05.

Inhibition of complement-mediated IRI has been shown to modulate a subsequent alloimmune response and to prolong graft survival in a VCA model when combined with a subtherapeutic dose of conventional immunosuppressant (22). We therefore evaluated the effect of B.PSel-Crry on the alloimmune response in terms of allograft survival after orthotopic hindlimb allotransplantation. A single postoperative 0.5mg dose of B.PSel-Crry prolonged allograft survival to a mean of 14 days, compared to a mean allograft survival of 10 days in vehicle treated recipients (**Figure 7**). Thus, B.PSel-Crry mediated protection against IRI translates to a modulated alloimmune response and increased graft survival.

## Discussion

Inappropriate activation of the complement system plays a major role in host cell injury in a number of inflammatory diseases and disease conditions. As such, complement activation pathways and complement effector products have long been recognized as potential druggable targets in the context of an anti-inflammatory therapeutic. While very few anti-complement therapeutics are currently approved, there are a multitude of complement inhibitors in clinical development and in clinical trials [reviewed in (26)]. The great majority of these inhibitors, including the approved inhibitors, cause and indeed require, systemic complement inhibition. However, complement plays important roles in multiple physiologic processes that include (but are not limited to) host defense, modulation of adaptive immune mechanisms, immune complex and cell debris removal, and tissue repair and regeneration. With this in mind, we have been investigating complement inhibitory strategies that block the pathological effects of complement activation while minimizing any effect on the physiological and beneficial roles of complement. Our general approach has been to target and localize complement inhibition to sites of disease and injury, and we have previously described two targeting strategies. One involved a targeting moiety consisting of a fragment of complement receptor 2 (CR2) that recognizes C3d deposited at sites of complement activation (23, 27), and the other an scFv that recognizes an injury specific neoepitope (13, 28). We have shown that targeted complement inhibitors, in contrast to their untargeted counterparts, have minimal effect on levels of systemic complement activity due to their short circulatory half-life, do not increase host susceptibility to infection, and by increasing bioavailability enhance efficacy up to 20-fold (23, 29). Here we describe a new class of targeted complement inhibitor in that one of the constructs characterized (B.Psel-Crry) is dual functioning with the targeting moiety itself directly contributing to protective activity *via* P-selectin blockade. This function of the targeting moiety manifests as reduced leukocyte recruitment to the injury site and improved IRI scores compared to NB.PSel-Crry, which targets to, but does not directly block P-selectin activity. We also show that the P-selectin targeted inhibitors have a short circulatory half-life, which will translate to a limited duration of any systemic inhibitory effects, and that they specifically target to an injured/transplanted limb to provide localized protection. To note, in the previous studies referenced above using targeted complement inhibitors (23, 29), lower doses of inhibitor were used in demonstrating that they had minimal effect on systemic complement inhibition and that they did not increase susceptibility to infection (0.1 mg for CR2-Crry and 0.2 mg for B4scFv-Crry). We cannot therefore rule out the possibility that the P-selectin constructs, at least for the higher 0.5 mg dose, had a more profound effect on systemic complement activity levels. It is worth noting, however, that the serum concentration of both B.Psel-Crry and NB.Psel-Crry is around 6 ug/ml at 15 min after injection of 0.5 mg. This likely reflects rapid tissue redistribution, and these serum concentrations are in line with previous studies using different complement inhibitors (13, 30, 31).

The P-selectin blocking construct provided improved protection in a hindlimb IRI model compared to the nonblocking construct, and this difference could not be accounted for in terms of differences in binding kinetics or *in vitro* complement inhibitory activity. There was also no difference in the level of deposited C3d between the two constructs, although reduced injury correlated with reduced C3d deposition for both constructs. There was, however, a greater inhibitory effect on myeloid cell recruitment with the blocking P-selectin construct, indicating that its enhanced protective activity is due to the function of the scFv targeting moiety. The nonblocking P-selectin construct reduced myeloid cell infiltration, although to a lesser extent than the blocking construct, but this is not surprising since there is a dynamic between P-selectin expression and complement activation; the expression of P-selectin can be upregulated by complement activation products and P-selectin can directly activate complement (5–9). Of note, endothelial expression of P-selectin has been shown to play a role in the recruitment of monocytes that secrete proangiogenic factors, and dysregulation of P-selectin has been shown to reduce vascular remodeling in limb ischemia (32). Thus, this represents an additional aspect by which P-selectin blockade may contribute to improved outcomes in our models.

An additional finding was that B.PSel-Crry also interfered with coagulation, as evidenced by increased bleeding time and volume in mice treated with the higher dose of the inhibitor, as well as improved tissue perfusion with B.PSel-Crry compared to NB.PSel-Crry in the hindlimb IRI model. The non-blocking construct had no effect on bleeding time/volume, although it did improve tissue perfusion at high dose, which is presumably *via* the effect of complement inhibition. Tissue perfusion was also improved in B.PSel-Crry treated animals following hindlimb transplantation compared to vehicle treated controls. In the context of the above findings, P-selectin plays important roles in platelet trafficking and coagulation, including coagulopathy and thrombosis (33), and complement is a major contributor to syndromes of thrombotic microangiopathy that display platelet aggregation and endothelial injury (34). Thus, the enhanced effect of dual P-selectin and complement blockade over complement inhibition alone suggests B.PSel-Crry (or humanized equivalent) may be particularly efficacious for treating certain coagulopathies. Relevant to the current study, microvascular thrombosis is also an important consideration in the transplant arena, and is a hallmark of xenograft rejection (35) and a major complication of hematopoietic stem cell transplantation (36). Of course, depending on the transplant setting, the B.Psel-Crry (but not NB.Psel-Crry), may also carry an increased risk of bleeding complications. In this context, the non-blocking P-selectin construct could also be a potential candidate for treating hemorrhagic conditions, such as hemorrhagic stroke/transformation in which complement inhibition has been shown to be protective (37, 38). To note, COVID-19 is also associated with a hypercoagulable state and hyper-complement activation, which may be involved in thrombotic microangiopathy and multi-organ complications (39). Although the focus here was on VCA transplants, it is likely B.PSel-Crry would be an equally suitable therapy in solid organ transplantation. Microvascular thrombosis is a common complication early and late post-transplantation (40–43), particularly in extended criteria donors, and thus application of B.PSel-Crry could represent a novel therapeutic intervention.

In terms of the specific disease conditions studied here, lower limb (warm) ischemia can occur during vascular surgery, following a thrombotic event, or following trauma. Vascularized composite allotransplantation is an emerging field, and there remain few studies examining VCA IRI and mitigation strategies. Donor VC allografts undergo brain death, a period of cold and warm ischemia, and they may be more susceptible to IRI as compared to solid organ grafts due to their heterogenous nature and multiple tissue types with varying immunogenicity (44). In the current study we show that B.Psel-Crry is similarly protective in both hindlimb IRI models utilized here, and that protection is associated with reduced complement activation and reduced myeloid cell infiltration. Of note, in the VCA model, protection from IRI translated to increased graft survival. Post-transplant IRI can promote acute rejection in experimental models, with the degree of early damage to the graft associated with an intensified rejection. Complement can also influence allorecognition, not only *via* its effect on post-transplant graft inflammation and IRI, but also *via* a direct effect of complement activation products on antigen presenting cells and lymphocytes. P-selectin is also an important mediator of leukocyte infiltration and injury (2). The infiltration of neutrophils in particular contributes to IRI, and adhesion molecules can also modulate the infiltration of lymphocytes involved in alloimmunity (45). Although little studied in the context of VCA, there is evidence that P-selectin plays a role in leukocyte extravasation in the skin, and selectin blockade inhibits leukocyte recruitment and injury (17, 46). P-selectin is also upregulated following human skin transplant and during rejection episodes (17).

It was previously shown that the decoration of soluble human CR1 (sCR1) with sialyl Lewis x (sLE x) moieties enhanced the protective effect of sCR1 in a rat model of selectin-dependent lung injury (47) and in a mouse model of ischemic stroke (48). The sLE x carbohydrate moiety binds to both P and E selectin. In the lung injury model, the enhanced protective effect of sCR1sLE x correlated with increased binding of sCR1sLe x to the lung vasculature when compared to binding of sCR1. sCR1sLE x also localized to the cerebral vasculature in the stroke model. Importantly, however, the efficacy of sCR1sLE x remained dependent on systemic complement inhibition. Furthermore, unlike the univalent scFv targeted constructs characterized here, sCR1sLE x contains polyvalent P-selectin binding sites, raising the possibility of homologous (platelet-platelet) and heterologous (platelet-endothelial cell) cross linking and platelet aggregation that may result in thrombotic complications.

To summarize, we describe two novel P-selectin targeted complement inhibitors, one of which also directly blocks P-selectin adhesion function and additionally interferes with coagulation. We demonstrate their effectiveness in two therapeutic paradigms of hindlimb injury, and have linked their different characteristics to protective outcomes. With regard to potential translation from the murine constructs characterized here, the Crry domain could be replaced with its human ortholog, CR1 (or fragment thereof). The anti-P-selectin scFv’s were derived from mAbs that recognize both mouse and human P-selectin (12), and we show that both scFv-Crry constructs bind both mouse and human P-selectin and would therefore only require humanization.

## Data Availability Statement

The datasets presented in this study can be found in online repositories. The names of the repository/repositories and accession number(s) can be found in the article/**Supplementary Material**.

## Ethics Statement

The animal study was reviewed and approved by MUSC IACUC Committee.

## Author Contributions

ST and CA designed and directed the study. ST directed writing of the manuscript. CZ, JR, QZ, and AA performed experiments. SN provided microsurgical assistance. XY generated the complement inhibitors and analyzed data. PE generated the anti-P-selectin mAbs from which the scFv’s were derived. JJ and SH analyzed the data and provided financial support. All authors reviewed and revised the manuscript. Each of the three first co-authors contributed an equal amount of time and effort to this work, and their position in the author list was decided based on the relative amount of their data shown in the manuscript.

## Funding

These studies were supported by the NIH (U01AI32894 and R56AI56383 to ST and CA), the Dept. Veteran’s Affairs (BX005235 to ST), the Dept. of Defense (RT190030 to ST, RW81XWH-16-1-0783 to CA), and an award from Enduring Hearts and the American Heart Association (18PRE34070023 to MS) and 111 Project from Ministry of Education and State Administration of Foreign Experts Affairs of the Peoples Republic of China (D17011 to ST, CA, and SH), and the Guangxi Distinguished Experts Special Fund (2019B12 to JJ).

## Conflict of Interest

The authors declare that the research was conducted in the absence of any commercial or financial relationships that could be construed as a potential conflict of interest.

## Publisher’s Note

All claims expressed in this article are solely those of the authors and do not necessarily represent those of their affiliated organizations, or those of the publisher, the editors and the reviewers. Any product that may be evaluated in this article, or claim that may be made by its manufacturer, is not guaranteed or endorsed by the publisher.

## Abbreviations

IR, ischemia reperfusion; IRI, ischemia reperfusion injury; Tx, transplantation; VC, vascularized composite; VCA, vascularized composite allotransplantation.

## References

[1] Olinic DM Stanek A Tataru DA Homorodean C Olinic M. Acute Limb Ischemia: An Update on Diagnosis and Management. J Clin Med (2019) 8(8). doi: 10.3390/jcm8081215 PMC6723825 31416204

[2] McEver RP. Selectins: Initiators of Leucocyte Adhesion and Signalling at the Vascular Wall. Cardiovasc Res (2015) 107(3):331–9. doi: 10.1093/cvr/cvv154 PMC4592324 25994174

[3] Gorsuch WB Chrysanthou E Schwaeble WJ Stahl GL. The Complement System in Ischemia-Reperfusion Injuries. Immunobiology (2012) 217(11):1026–33. doi: 10.1016/j.imbio.2012.07.024 PMC3439809 22964228

[4] Arumugam TV Magnus T Woodruff TM Proctor LM Shiels IA Taylor SM. Complement Mediators in Ischemia-Reperfusion Injury. Clin Chim Acta (2006) 374(1-2):33–45. doi: 10.1016/j.cca.2006.06.010 16872589

[5] Lozada C Levin RI Huie M Hirschhorn R Naime D Whitlow M. Identification of C1q as the Heat-Labile Serum Cofactor Required for Immune Complexes to Stimulate Endothelial Expression of the Adhesion Molecules E-Selectin and Intercellular and Vascular Cell Adhesion Molecules 1. Proc Natl Acad Sci USA (1995) 92:8378–82. doi: 10.1073/pnas.92.18.8378 PMC41160 7545301

[6] Hattori R Hamilton KK McEver RP Sims PJ. Complement Proteins C5b-9 Induce Secretion of High Molecular Weight Multimers of Endothelial Von Willebrand Factor and Translocation of Granule Membrane Protein GMP-140 to the Cell Surface. J Biol Chem (1989) 264:7768–71. doi: 10.1016/S0021-9258(18)83104-0 2470750

[7] Foreman KE Vaporciyan AA Bonish BK Jones ML Johnson KJ Glovsky MM. C5a-Induced Expression of P-Selectin in Endothelial Cells. J Clin Invest (1994) 94:1147–55. doi: 10.1172/JCI117430 PMC295185 7521884

[8] Atkinson C Zhu H Qiao F Varela JC Yu J Song H. Complement-Dependent P-Selectin Expression and Injury Following Ischemic Stroke. J Immunoly (2006) 177(10):7266–74. doi: 10.4049/jimmunol.177.10.7266 17082645

[9] Del Conde I Cruz MA Zhang H Lopez JA Afshar-Kharghan V. Platelet Activation Leads to Activation and Propagation of the Complement System. J Exp Med (2005) 201(6):871–9. doi: 10.1084/jem.20041497 PMC2213112 15781579

[10] Kim YU Kinoshita T Molina H Hourcade D Seya T Wagner LM. Mouse Complement Regulatory Protein Crry/p65 Uses the Specific Mechanisms of Both Human Decay-Accelerating Factor and Membrane Cofactor Protein. J Exp Med (1995) 181:151–9. doi: 10.1084/jem.181.1.151 PMC2191854 7528766

[11] Amersdorfer P Wong C Chen S Smith T Deshpande S Sheridan R. Molecular Characterization of Murine Humoral Immune Response to Botulinum Neurotoxin Type A Binding Domain as Assessed by Using Phage Antibody Libraries. Infect Immun (1997) 65(9):3743–52. doi: 10.1128/iai.65.9.3743-3752.1997 PMC175534 9284147

[12] Massaguer A Engel P Perez-del-Pulgar S Bosch J Pizcueta P. Production and Characterization of Monoclonal Antibodies Against Conserved Epitopes of P-Selectin (CD62P). Tissue Antigens (2000) 56(2):117–28. doi: 10.1034/j.1399-0039.2000.560202.x 11019911

[13] Atkinson C Qiao F Yang X Zhu P Reaves N Kulik L. Targeting Pathogenic Postischemic Self-Recognition by Natural IgM to Protect Against Posttransplantation Cardiac Reperfusion Injury. Circulation (2015) 131(13):1171–80. doi: 10.1161/CIRCULATIONAHA.114.010482 PMC4382916 25825397

[14] Quigg RA Kozono Y Berthiaume D Lim A Salant J Weinfeld A. Blockade of Antibody-Induced Glomerulonephritis With Crry-Ig, a Soluble Murine Complement Inhibitor. J Immunol (1998) 160:4553–60. 9574562

[15] Crawford RS Hashmi FF Jones JE Albadawi H McCormack M Eberlin K. A Novel Model of Acute Murine Hindlimb Ischemia. Am J Physiol Heart Circ Physiol (2007) 292(2):H830–7. doi: 10.1152/ajpheart.00581.2006 17012358

[16] Furtmuller GJ Oh B Grahammer J Lin CH Sucher R Fryer ML. Orthotopic Hind Limb Transplantation in the Mouse. J Vis Exp JoVE (2016) 108):53483. doi: 10.3791/53483 PMC4828154 26967527

[17] Hautz T Zelger B Grahammer J Krapf C Amberger A Brandacher G. Molecular Markers and Targeted Therapy of Skin Rejection in Composite Tissue Allotransplantation. Am J Transpl (2010) 10(5):1200–9. doi: 10.1111/j.1600-6143.2010.03075.x 20353468

[18] Wyseure T Cooke EJ Declerck PJ Behrendt N Meijers JCM von Drygalski A. Defective TAFI Activation in Hemophilia A Mice Is a Major Contributor to Joint Bleeding. Blood (2018) 132(15):1593–603. doi: 10.1182/blood-2018-01-828434 PMC6182268 30026184

[19] McCormack MC Kwon E Eberlin KR Randolph M Friend DS Thomas AC. Development of Reproducible Histologic Injury Severity Scores: Skeletal Muscle Reperfusion Injury. Surgery (2008) 143(1):126–33. doi: 10.1016/j.surg.2007.06.005 18154940

[20] Atkinson C Varela JC Tomlinson S. Complement-Dependent Inflammation and Injury in a Murine Model of Brain Dead Donor Hearts. Circ Res (2009) 105(11):1094–101. doi: 10.1161/CIRCRESAHA.109.194977 PMC2783176 19815824

[21] Oklu R Albadawi H Jones JE Yoo HJ Watkins MT. Reduced Hind Limb Ischemia-Reperfusion Injury in Toll-Like Receptor-4 Mutant Mice Is Associated With Decreased Neutrophil Extracellular Traps. J Vasc Surg (2013) 58(6):1627–36. doi: 10.1016/j.jvs.2013.02.241 PMC3825746 23683381

[22] Zhu P Bailey SR Lei B Paulos CM Atkinson C Tomlinson S. Targeted Complement Inhibition Protects Vascularized Composite Allografts From Acute Graft Injury and Prolongs Graft Survival When Combined With Subtherapeutic Cyclosporine A Therapy. Transplantation (2017) 101(4):e75–85. doi: 10.1097/TP.0000000000001625 PMC5360489 28045880

[23] Atkinson C Song H Lu B Qiao F Burns TA Holers VM. Targeted Complement Inhibition by C3d Recognition Ameliorates Tissue Injury Without Apparent Increase in Susceptibility to Infection. J Clin Invest (2005) 115(9):2444–53. doi: 10.1172/JCI25208 PMC1190375 16127466

[24] Kyriakides C Austen W Jr. Wang Y Favuzza J Kobzik L Moore FD Jr.. Skeletal Muscle Reperfusion Injury is Mediated by Neutrophils and the Complement Membrane Attack Complex. Am J Physiol (1999) 277(6):C1263–8. doi: 10.1152/ajpcell.1999.277.6.C1263 10600778

[25] Kyriakides C Austen WG Jr. Wang Y Favuzza J Moore FD Jr Hechtman HB. Neutrophil Mediated Remote Organ Injury After Lower Torso Ischemia and Reperfusion is Selectin and Complement Dependent. J Trauma (2000) 48(1):32–8. doi: 10.1097/00005373-200001000-00006 10647562

[26] Zelek WM Xie L Morgan BP Harris CL. Compendium of Current Complement Therapeutics. Mol Immunol (2019) 114:341–52. doi: 10.1016/j.molimm.2019.07.030 31446305

[27] Song H He C Knaak C Guthridge JM Holers VM Tomlinson S. Complement Receptor 2-Mediated Targeting of Complement Inhibitors to Sites of Complement Activation. J Clin Invest (2003) 111(12):1875–85. doi: 10.1172/JCI17348 PMC161422 12813023

[28] Li C Patel K Tu Z Yang X Kulik L Alawieh A. A Novel Injury Site-Natural Antibody Targeted Complement Inhibitor Protects Against Lung Transplant Injury. Am J Transpl (2021) 21:2067–78. doi: 10.1111/ajt.16404 PMC8246004 33210808

[29] Alawieh A Langley EF Tomlinson S. Targeted Complement Inhibition Salvages Stressed Neurons and Inhibits Neuroinflammation After Stroke in Mice. Sci Trans Med (2018) 10(441)179–86. doi: 10.1126/scitranslmed.aao6459 PMC6689196 29769288

[30] Huang Y Qiao F Atkinson C Holers VM Tomlinson S. A Novel Targeted Inhibitor of the Alternative Pathway of Complement and Its Therapeutic Application in Ischemia/Reperfusion Injury. J Immunol (2008) 181(11):8068–76. 10.4049/jimmunol.181.11.8068 PMC2782395 19017999

[31] Zheng C Sleiman MM Yang X He S Atkinson C Tomlinson S. Increasing the Efficacy and Safety of a Human Complement Inhibitor for Treating Post-Transplant Cardiac Ischemia Reperfusion Injury by Targeting to a Graft-Specific Neoepitope. J Heart Lung Transplant (2021) 40(10):1112–21. doi: 10.1016/j.healun.2021.07.004 PMC10587835 34334299

[32] Carr CL Qi Y Davidson B Chadderdon S Jayaweera AR Belcik JT. Dysregulated Selectin Expression and Monocyte Recruitment During Ischemia-Related Vascular Remodeling in Diabetes Mellitus. Arterioscler Thromb Vasc Biol (2011) 31(11):2526–33. doi: 10.1161/ATVBAHA.111.230177 PMC3202967 21885854

[33] Cambien B Wagner DD. A New Role in Hemostasis for the Adhesion Receptor P-Selectin. Trends Mol Med (2004) 10(4):179–86. doi: 10.1016/j.molmed.2004.02.007 15059609

[34] Gavriilaki E Anagnostopoulos A Mastellos DC. Complement in Thrombotic Microangiopathies: Unraveling Ariadne’s Thread Into the Labyrinth of Complement Therapeutics. Front Immunol (2019) 10:337. doi: 10.3389/fimmu.2019.00337 30891033 PMC6413705

[35] Rosales IA Colvin RB. The Pathology of Solid Organ Xenotransplantation. Curr Opin Organ Transpl (2019) 24(5):535–42. doi: 10.1097/MOT.0000000000000681 31348015

[36] Gavriilaki E Sakellari I Anagnostopoulos A Brodsky RA. Transplant-Associated Thrombotic Microangiopathy: Opening Pandora’s Box. Bone Marrow Transplant (2017) 52(10):1355–60. doi: 10.1038/bmt.2017.39 28287636

[37] Garrett MC Otten ML Starke RM Komotar RJ Magotti P Lambris JD. Synergistic Neuroprotective Effects of C3a and C5a Receptor Blockade Following Intracerebral Hemorrhage. Brain Res (2009) 1298:171–7. doi: 10.1016/j.brainres.2009.04.047 PMC2760685 19410563

[38] Alawieh A Chalhoub RM Mallah K Langley EF York M Broome H. Complement Drives Synaptic Degeneration and Progressive Cognitive Decline in the Chronic Phase After Traumatic Brain Injury. J Neurosci (2021) 41(8):1830–43. doi: 10.1523/JNEUROSCI.1734-20.2020 PMC8115878 33446516

[39] Ghebrehiwet B Peerschke EI. Complement and Coagulation: Key Triggers of COVID-19-Induced Multiorgan Pathology. J Clin Invest (2020) 130(11):5674–6. doi: 10.1172/JCI142780 PMC7598033 32925166

[40] Hessheimer AJ Vendrell M Munoz J Ruiz A Diaz A Siguenza LF. Heparin But Not Tissue Plasminogen Activator Improves Outcomes in Donation After Circulatory Death Liver Transplantation in a Porcine Model. Liver Transpl (2018) 24(5):665–76. doi: 10.1002/lt.25013 29351369

[41] Sartain S Shubert S Wu MF Wang T Martinez C. The Alternative Complement Pathway Activation Product Ba as a Marker for Transplant-Associated Thrombotic Microangiopathy. Pediatr Blood Cancer (2020) 67(3):e28070. doi: 10.1002/pbc.28070 31774252

[42] Bjerre KP Clemmensen TS Berg K Poulsen SH Hvas AM Grove EL. Platelet Aggregation and Response to Aspirin Therapy in Cardiac Allograft Vasculopathy. J Heart Lung Transplant (2020) 39(4):371–8. doi: 10.1016/j.healun.2020.01.1344 32067865

[43] Kalla S Ellis RJ Campbell SB Doucet B Isbel N Tie B. Thrombotic Microangiopathy Associated With Pazopanib in a Kidney Transplant Recipient. J Kidney Cancer VHL (2021) 8(1):25–31. doi: 10.15586/jkcvhl.v8i1.161 33850692 PMC8017890

[44] Caterson EJ Lopez J Medina M Pomahac B Tullius SG. Ischemia-Reperfusion Injury in Vascularized Composite Allotransplantation. J Craniofac Surg (2013) 24(1):51–6. doi: 10.1097/SCS.0b013e31827104e1 23321872

[45] Jones TR Shirasugi N Adams AB Pearson TC Larsen CP. Intravital Microscopy Identifies Selectins That Regulate T Cell Traffic Into Allografts. J Clin Invest (2003) 112(11):1714–23. doi: 10.1172/JCI19391 PMC281648 14660747

[46] Friedrich M Bock D Philipp S Ludwig N Sabat R Wolk K. Pan-Selectin Antagonism Improves Psoriasis Manifestation in Mice and Man. Arch Dermatol Res (2006) 297(8):345–51. doi: 10.1007/s00403-005-0626-0 16362415

[47] Mulligan MS Warner RL Rittershaus CW Thomas LJ Ryan US Foreman KE. Endothelial Targeting and Enhanced Antiinflammatory Effects of Complement Inhibitors Possessing Sialyl Lewisx Moieties. J Immunol (1999) 162(8):4952–9. 10202042

[48] Huang J Kim LJ Mealey R Marsh HC Jr Zhang Y Tenner AJ. Neuronal Protection in Stroke by an Slex-Glycosylated Complement Inhibitory Protein. Science (1999) 285(5427):595–9. doi: 10.1126/science.285.5427.595 10417391

