# Supplementary material for: Characterization of Novel P-Selectin Targeted Complement Inhibitors in Murine Models of Hindlimb Injury and Transplantation
Source: Front Immunol. 2021 Nov 25;12:785229. doi: 10.3389/fimmu.2021.785229 (PMC8654931; doi:10.3389/fimmu.2021.785229)
Supplement: Supplementary file 1 [file DataSheet_1.docx]

Supplementary Material

## Supplementary Figures

**
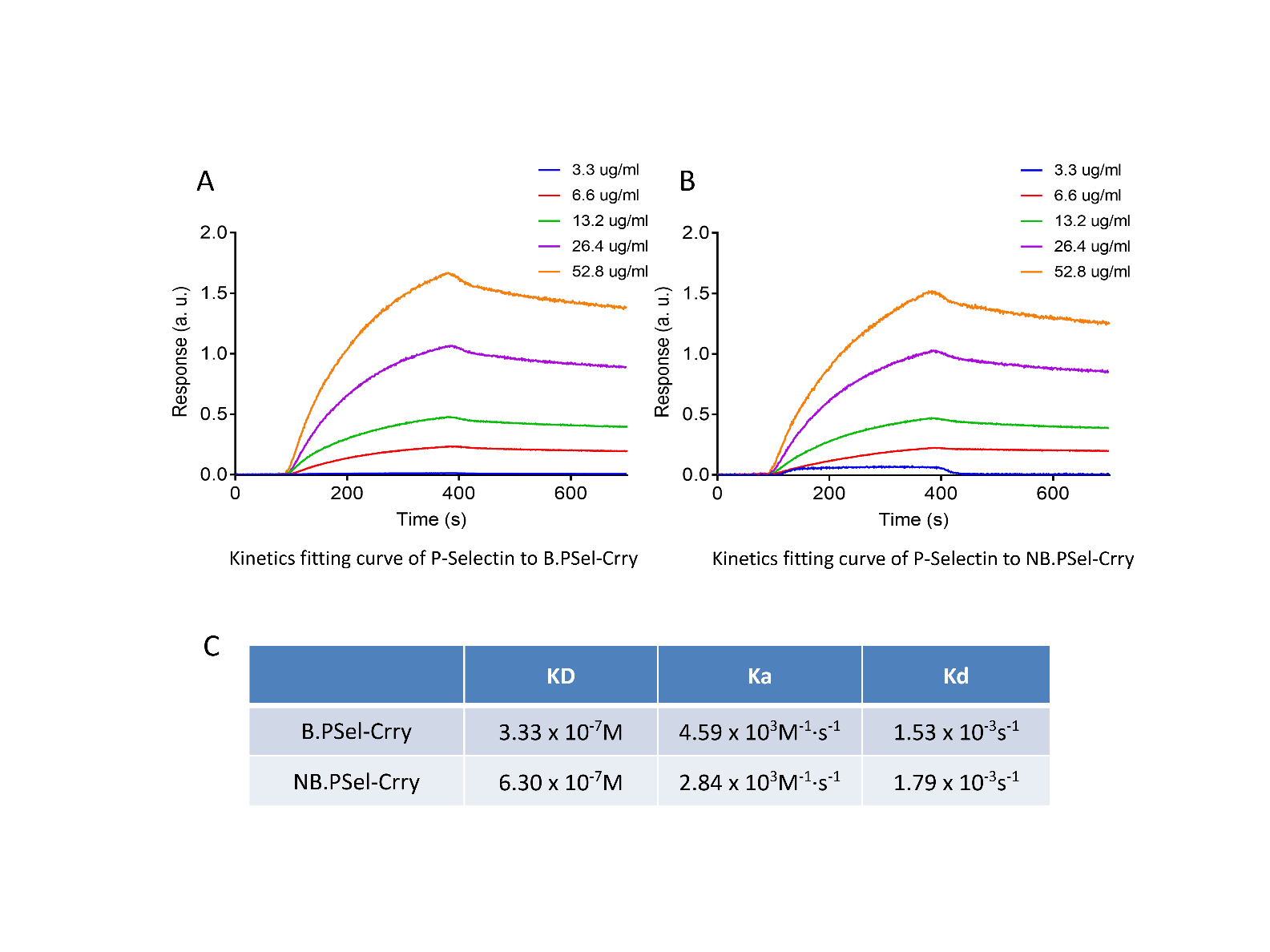
**

**Supplementary figure 1.** **Binding kinetics of B.Psel-Crry and NB. Psel-Crry to mouse P-selectin**. Surface plasmon resonance determination of binding affinity KD and kinetic parameters ka, kd of constructs. Ligand is mouse P-Selectin-His tag, and analytes are B.Psel-Crry and NB. Psel-Crry. A**.** Kinetics curves of B.Psel-Crry. B. Kinetics curves of NB.Psel-Crry. C. Calculated equilibrium dissociation constant (KD), association rate constant (Ka) and dissociation rate constant (Kd).

**
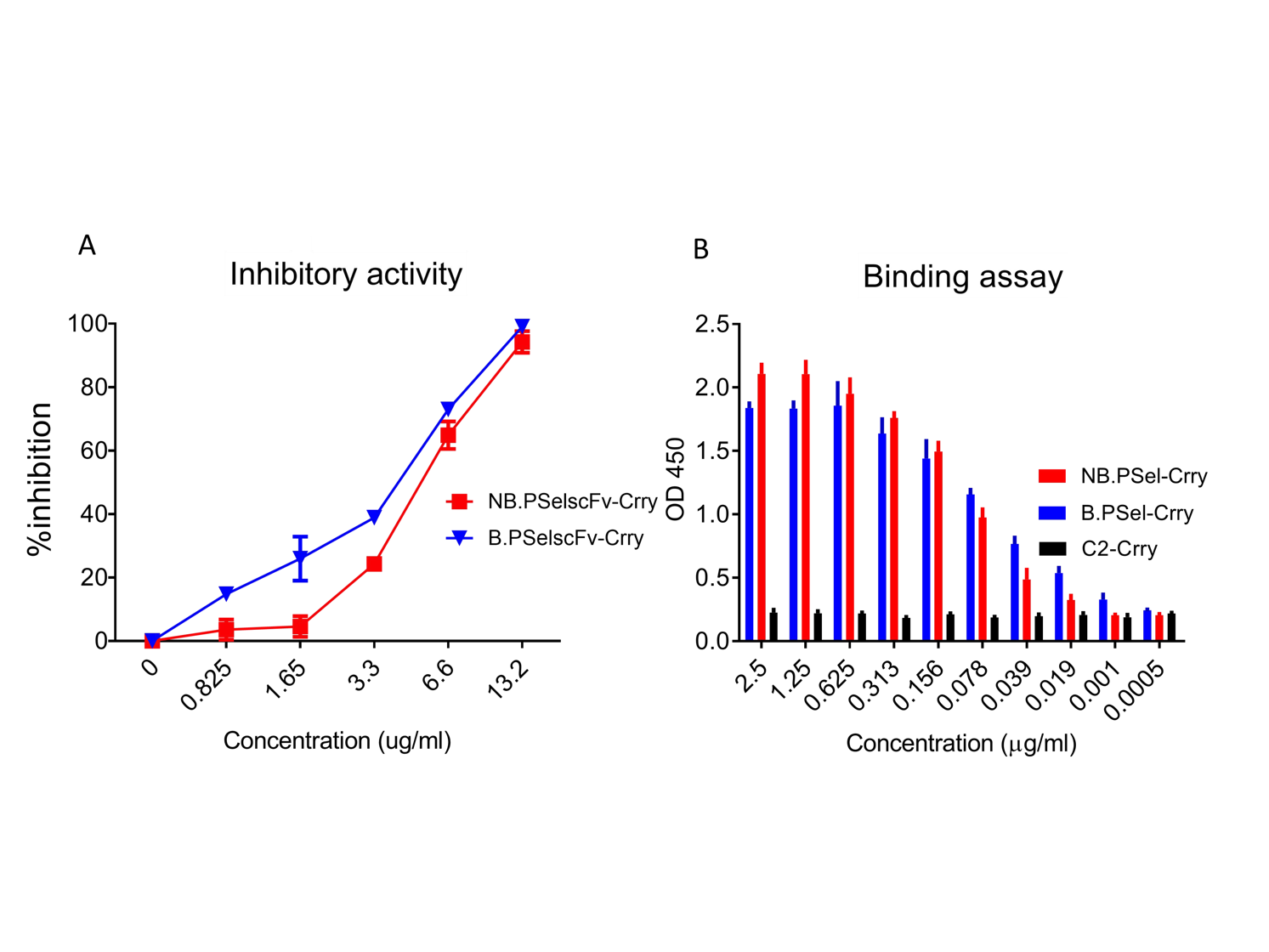
**

**Supplementary** **figure 2. In vitro complement inhibitory and human P-selectin binding activity of B.PSel-Crry and NB.PSel-Crry**. A. Complement inhibitory activity as determined by C3d deposition on zymosan beads. Mean +/- SD, p > 0.05. n = 3. B. Dose-dependent binding of both constructs as determined by ELISA using human -selectin coated plates. C2-Crry is a scFv-Crry fusion protein in which the C2scFv moiety targets a subset of phospholipids (45). Mean +/- SD, n = 3.


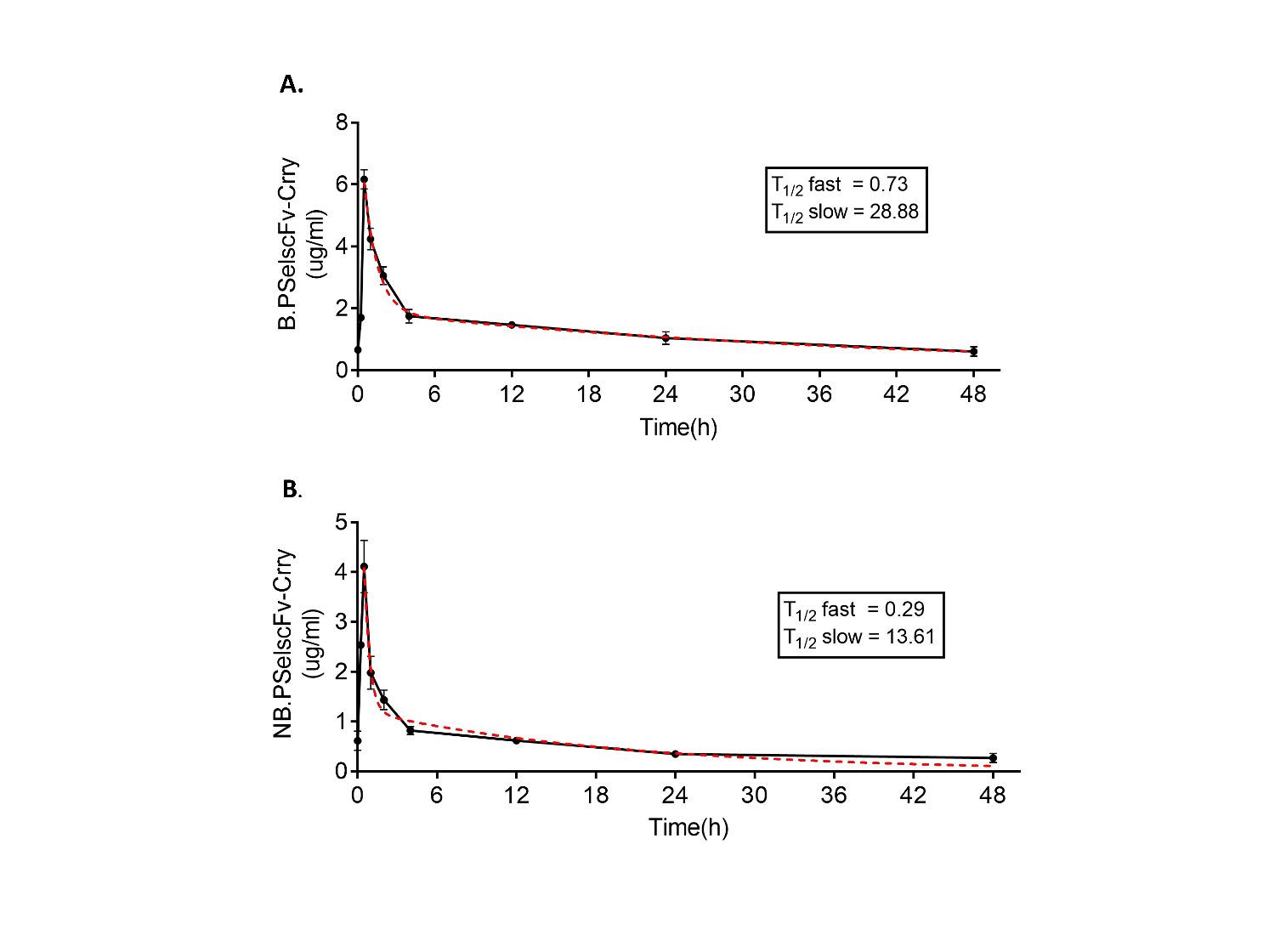


**Supplementary** **figure 3. Circulatory half-life determination of B.Psel-Crry and NB.Psel-Crry.** The serum levels of each construct were measured by ELISA in serum prepared from blood collected at indicated times following tail vein injection (0.5 mg). Mean ± SD; n = 3.
